# Dengue Outbreak Response during COVID-19 Pandemic, Key Largo, Florida, USA, 2020

**DOI:** 10.3201/eid2908.221856

**Published:** 2023-08

**Authors:** Devin Rowe, Catherine McDermott, Ysla Veliz, Alison Kerr, Mark Whiteside, Mikki Coss, Chad Huff, Andrea Leal, Edgar Kopp, Alexis LaCrue, Lea A. Heberlein, Laura E. Adams, Gilberto A. Santiago, Jorge L. Munoz-Jordan, Gabriela Paz-Bailey, Andrea M. Morrison, Florida Department of Health Dengue Investigation Team

**Affiliations:** Florida Department of Health, Tallahassee, Florida, USA (D. Rowe, C. McDermott, Y. Veliz, A. Kerr, M. Whiteside, E. Kopp, A. LaCrue, L.A. Heberlein, A.M. Morrison);; Florida Keys Mosquito Control District, Marathon, Florida, USA (M. Coss, C. Huff, A. Leal);; Centers for Disease Control and Prevention, San Juan, Puerto Rico, USA (L.E. Adams, G.A. Santiago, J.L. Munoz-Jordan, G. Paz-Bailey)

**Keywords:** Dengue, COVID-19, dengue virus, viruses, respiratory infections, zoonoses, vector-borne infections, DENV, severe acute respiratory syndrome coronavirus 2, SARS-CoV-2, coronavirus disease, Florida, United States

## Abstract

We report a dengue outbreak in Key Largo, Florida, USA, from February through August 2020, during the COVID-19 pandemic. Successful community engagement resulted in 61% of case-patients self-reporting. We also describe COVID-19 pandemic effects on the dengue outbreak investigation and the need to increase clinician awareness of dengue testing recommendations.

Dengue, an arboviral disease caused by dengue viruses 1–4 (DENV-1–4), is transmitted by *Aedes aegypti* mosquitoes (*1*). Before 1935, dengue was endemic in Florida, USA (*2*); however, no locally acquired cases were reported until an outbreak in Key West during 2009–2010 (*3*). Since then, at least 1 locally transmitted DENV infection has been reported annually in Florida except for 2017 and 2021 (*2*). Because Florida is vulnerable to establishment of *Ae. aegypti*–vectored viruses such as dengue, chikungunya, and Zika (*4*,*5*), surveillance is crucial to detect pathogen introduction.

During the COVID-19 pandemic, detecting the cause for other febrile illnesses was challenging (*6*) and reluctance to seek medical care during the pandemic was reported (*7*). We report the response to a dengue outbreak in Florida during the COVID-19 pandemic in 2020.

## The Study

**Figure 1:**
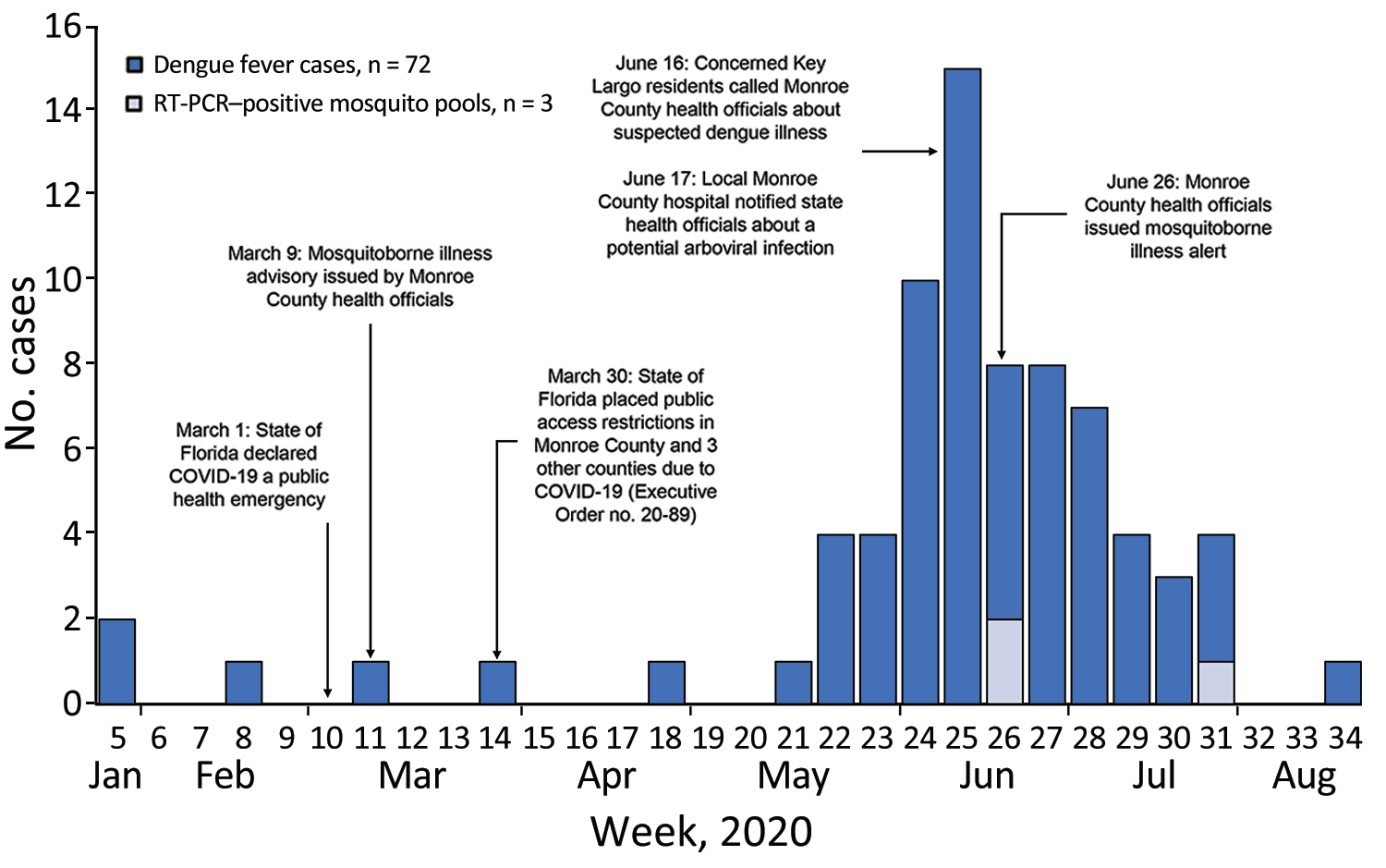
Timeline of dengue outbreak response during COVID-19 pandemic, Key Largo, Florida, USA, 2020. Timeline shows number of dengue cases, dengue virus RT-PCR–positive mosquito pools, and events per week during January 26–August 20, 2020. RT-PCR, reverse transcription PCR.

On February 28, 2020, the Florida Department of Health (FDOH) was notified of a possible locally acquired dengue case in a non-Florida resident who was visiting Key Largo; the case-patient had symptom onset on February 18. After confirming DENV-1 infection, FDOH issued a countywide public health mosquitoborne illness advisory for Monroe County on March 9 (Figure 1). During that same month, the governor of Florida issued a statewide public health emergency declaration for the COVID-19 pandemic (*8*). By the end of March, public access to nonessential businesses and facilities was further restricted in Monroe County because of increased COVID-19 case numbers (*9*). Additional locally acquired dengue cases were not identified until June 16, when several concerned Key Largo residents called FDOH reporting suspected dengue illness. A mosquitoborne illness alert was subsequently issued for the county after 8 local dengue cases were confirmed.

FDOH notified the Florida Keys Mosquito Control District (FKMCD) of possible mosquito exposure locations for suspected cases during the 2-week incubation period through the potential 1-week viremic period after symptom onset. FKMCD enhanced aerial and truck spraying and canvassed neighborhoods to conduct vector surveillance, remove or treat mosquito larval habitats, and provide mosquito control education. 

While also responding to COVID-19, FDOH fielded hotline calls for residents reporting dengue-like illness, interviewed suspected case-patients, conducted site visits, provided frequent healthcare provider and community outreach, collected serum samples for DENV testing, and promptly provided updates to FKMCD and local media. Persons with suspected dengue were asked to provide contact information for other persons who shared mosquito exposure risks, such as persons from the same household, workplace, or outdoor events. FDOH reached out to contacts and offered DENV testing if they reported a recent unexplained febrile illness. Ethics approval was not required because the activities conducted were part of standard public health outbreak surveillance and response.

FDOH also conducted syndromic surveillance for chief complaint and discharge diagnosis records from local hospitals. FDOH reviewed all syndromic surveillance records in the primary hospital serving the outbreak area and countywide, prioritizing chief complaints and discharge diagnoses mentioning dengue or fever and any combination of thrombocytopenia, rash, or arthralgia. FDOH requested medical records for patient visits with no alternative diagnosis. If only dengue serology had been ordered, FDOH requested that specimens be forwarded to the state laboratory for reverse transcription PCR (RT-PCR) testing. If no alternative diagnosis had been made and no DENV testing previously ordered, FDOH offered testing for persons with suspected cases.

Consistent with Centers for Disease Control and Prevention (CDC) guidelines, FDOH tested acute specimens collected within 7 days after symptom onset by using DENV RT-PCR and IgM tests. We only routinely performed antibody testing on convalescent samples collected >7 days after symptom onset. Specimens with positive or equivocal DENV test results at commercial laboratories were forwarded to FDOH and similarly retested. CDC assisted with serologic confirmation, serotyping RT-PCR–positive samples, and provided RT-PCR testing for mosquito pools collected by FKMCD.

We identified 72 locally acquired dengue cases associated with Key Largo. Cases were primarily among female (51%) and non-Hispanic (83%) persons (Table). Self-reporting, including via contact outreach, drove initial case identification (61%), followed by commercial laboratory reporting (22%), and syndromic surveillance (7%); only 1 case was first identified through direct healthcare provider reporting. No case-patients had traveled outside the continental United States during the incubation period.

**Table:** Characteristics of case-patients in a dengue outbreak during the COVID-19 pandemic, Key Largo, Florida, USA, 2020*

Characteristics	No. (%) cases, n = 72
Sex	
F	37 (51)
M	35 (49)
Ethnicity	
Non-Hispanic	60 (83)
Hispanic	10 (14)
Unknown	2 (3)
Age group, y	
0–20	8 (11)
21–40	9 (13)
41–60	38 (53)
>60	17 (24)
Hospitalization status	
Hospitalized	8 (11)
Not hospitalized	62 (86)
Unknown	2 (3)
Laboratory test results†	
Positive RT-PCR	31 (43)
Positive IgM only	41 (57)
Tests ordered for acute dengue cases, n = 43‡	
COVID-19 tests; no known DENV tests	25 (58)
DENV and COVID-19 tests	13 (30)
DENV only; no COVID-19 tests	3 (7)
No DENV or COVID-19 tests	2 (5)

*DENV, dengue virus; RT-PCR, reverse transcription PCR. †Samples for PCR collected 1–12 days after symptom onset; samples for IgM collected 5–213 days after symptom onset. ‡Acute cases were tested within 7 days after symptom onset. Data include only persons medically evaluated by a healthcare provider in the United States. Information on care-seeking behavior was determined through case interview and laboratory records. Negative COVID-19 results were required to be reported to Florida Department of Health, but negative dengue laboratory results are not, which might have resulted in underreporting of commercial dengue testing among persons who sought care.

Overall, 31 cases were RT-PCR–positive and 41 were IgM-positive (Table). All RT-PCR–positive cases were DENV-1. Retrospective case finding and testing identified IgM-positive cases with reported symptom onset as early as January (Figure 1). Some persons identified through retrospective case finding reported a febrile illness several months prior. We presumed those febrile illnesses were dengue, but asymptomatic infections are common, and IgM is generally detectable for 3 months, making definitive confirmation of the timing of DENV infection difficult. 

**Figure 2:**
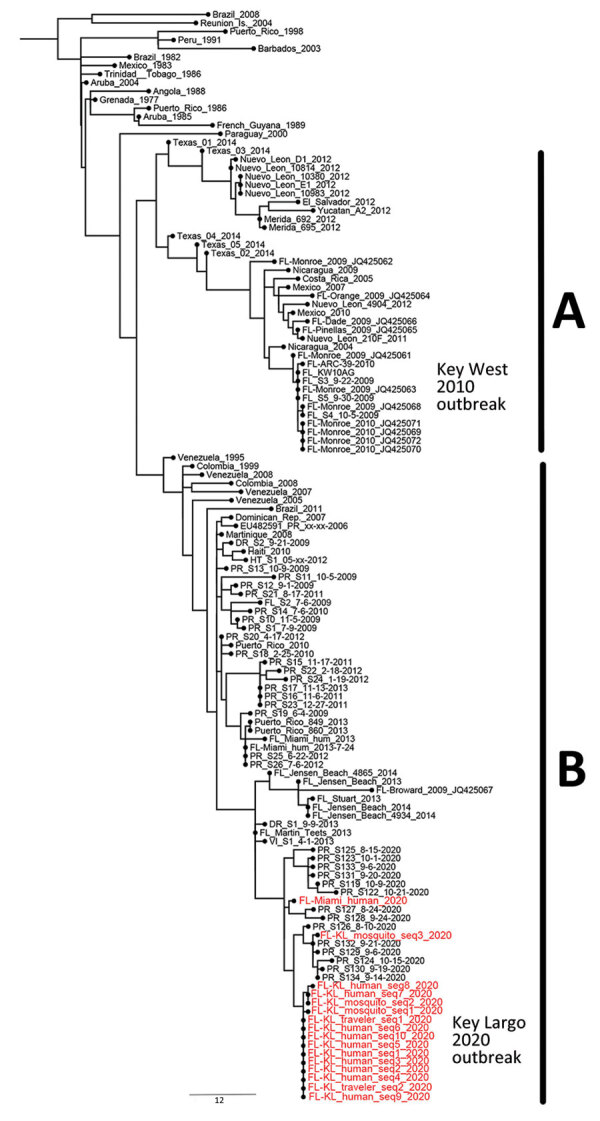
Phylogenetic reconstruction of dengue virus 1 from a dengue outbreak response during COVID-19 pandemic, Key Largo, Florida, USA, 2020. A) Central American lineage, 1986–2014; B) Caribbean lineage, 2008–2020. Maximum-likelihood tree of genotype V was inferred by using envelope gene sequences representing the Central American and Caribbean lineages. Red text indicates sequences obtained in this study. Sequence FL-Miami_human_2020 was obtained from a Miami-Dade County resident with recent travel history to Cuba. We obtained 2 sequences (GenBank accession nos. OM909246 and OM909247) from the National Reference Laboratory for Arboviruses, French Armed Forces Biomedical Research Institute, Bretigny-sur-Orge, France. Scale bar indicates nucleotide substitutions per site.

Among 96 *Ae. aegypti* mosquito pools collected during June 18–September 21, three tested positive for DENV-1 (Figure 1). We sequenced 15 positive samples, 12 from dengue cases and 3 from mosquito pools. Phylogenetic analysis showed grouping within the Caribbean lineage of DENV-1 genotype V (Figure 2). Sequences from mosquito pools and humans were almost identical. We published sequence data in GenBank (accession nos. OM831209–18, OM833055–59, and OM909246–47). Sequencing definitively differentiated this outbreak of Caribbean lineage DENV from the 2009–2010 Key West outbreak of Central American lineage.

Among case-patients, 43 (60%) visited a healthcare provider during the acute illness, within 1 week after symptom onset (Table). Providers considered COVID-19 as a potential diagnosis, which is evidenced by COVID-19 test orders for 38 (88%) of the acute dengue cases. Dengue was considered a potential diagnosis in only 16 acute cases, 13 of which had testing for both dengue and COVID-19. Providers primarily (75%) ordered dengue antibody testing when evaluating acute cases, which is inconsistent with CDC recommendations to use RT-PCR or DENV nonstructural protein 1 (NS1) test (*10*), an alternative to RT-PCR, in addition to IgM testing during the acute phase. No acute samples were tested using the DENV NS1 test. Among acute samples, 26 had comprehensive testing (both RT-PCR and IgM) performed at a reference laboratory at FDOH or CDC. Eight were positive for both assays, 14 were only DENV RT-PCR–positive, and 4 were only DENV IgM-positive. Ultimately, 54% of acute cases tested with only an IgM assay would have been missed if not for additional RT-PCR testing performed at a reference laboratory, compared with just 15% missed by using RT-PCR testing alone.

## Conclusions

This investigation confirmed an ongoing dengue outbreak in the Key Largo area of Florida, USA, during January–August 2020. During that same timeframe, 1,692 COVID-19 cases were reported in Monroe County. We suspect the COVID-19 pandemic negatively affected dengue surveillance because of reluctance to seek medical care, competing demands on healthcare providers during a rapidly evolving pandemic, and similar clinical presentations between COVID-19 and dengue. The focus on COVID-19 was further evidenced by providers primarily ordering COVID-19 tests among patients with acute dengue seeking medical care. The use of multiple case-finding methods, including aggressive community engagement, helped mitigate some of those effects, as did pandemic-related travel restrictions in the county. 

In conclusion, CDC recommends using either commercial DENV RT-PCR or NS1 tests in combination with serologic testing for samples collected during the acute phase of dengue illness (*10*). This outbreak highlights that those tests were underused. Improving clinician awareness of CDC recommendations could improve case detection in the future, especially for nonendemic areas at increased risk for DENV introduction.

## References

[1] Gubler DJ. Dengue and dengue hemorrhagic fever. Clin Microbiol Rev. 1998;11:480–96. 10.1128/CMR.11.3.480 9665979 PMC88892

[2] Florida Department of Health. Mosquito-borne disease surveillance [cited 2021 Dec 31]. https://www.floridahealth.gov/diseases-and-conditions/mosquito-borne-diseases/surveillance.html

[3] Trout A, Baracco G, Rodriguez M, Barber J, Leal A, Radke E, et al.; Centers for Disease Control and Prevention (CDC). Locally acquired Dengue—Key West, Florida, 2009-2010. MMWR Morb Mortal Wkly Rep. 2010;59:577–81. 20489680

[4] Kendrick K, Stanek D, Blackmore C; Centers for Disease Control and Prevention (CDC). Notes from the field: Transmission of chikungunya virus in the continental United States—Florida, 2014. MMWR Morb Mortal Wkly Rep. 2014;63:1137. 25474035 PMC4584604

[5] Likos A, Griffin I, Bingham AM, Stanek D, Fischer M, White S, et al. Local Mosquito-Borne Transmission of Zika Virus - Miami-Dade and Broward Counties, Florida, June-August 2016. MMWR Morb Mortal Wkly Rep. 2016;65:1032–8. 10.15585/mmwr.mm6538e1 27684886

[6] Centers for Disease Control and Prevention. Is it dengue or is it COVID-19? [cited 2020 Dec 11]. https://www.cdc.gov/dengue/healthcare-providers/dengue-or-covid.html

[7] Czeisler MÉ, Marynak K, Clarke KEN, Salah Z, Shakya I, Thierry JM, et al. Delay or avoidance of medical care because of COVID-19–related concerns—United States, June 2020. MMWR Morb Mortal Wkly Rep. 2020;69:1250–7. 10.15585/mmwr.mm6936a4 32915166 PMC7499838

[8] State of Florida. Executive order number 20-51. Establishes COVID-19 response protocol and directs public health emergency [cited 2021 Dec 31]. https://www.flgov.com/wp-content/uploads/orders/2020/EO_20-51.pdf

[9] State of Florida Exec. Executive order number 20-89. Emergency management—COVID-19—Miami-Dade County, Broward County, Palm Beach County, Monroe County public access restrictions [cited 2021 Dec 31]. https://www.flgov.com/wp-content/uploads/orders/2020/EO_20-89.pdf

[10] US Centers for Disease Control and Prevention. Dengue testing guidance for healthcare providers [cited 2020 Dec 11]. https://www.cdc.gov/dengue/healthcare-providers/testing/testing-guidance.html

